# Rad51 Expression Is a Useful Predictive Factor for the Efficacy of Neoadjuvant Chemoradiotherapy in Squamous Cell Carcinoma of the Esophagus

**DOI:** 10.1245/s10434-013-3220-2

**Published:** 2013-09-25

**Authors:** Tomonori Nakanoko, Hiroshi Saeki, Masaru Morita, Yuichiro Nakashima, Koji Ando, Eiji Oki, Takefumi Ohga, Yoshihiro Kakeji, Yasushi Toh, Yoshihiko Maehara

**Affiliations:** 1 Department of Surgery and Science, Graduate School of Medical Sciences, Kyushu University, Fukuoka, Japan; 2 Department of Gastrointestinal Surgery, National Kyushu Cancer Center, Fukuoka, Japan

## Abstract

**Background:**

Neoadjuvant chemoradiotherapy (NACRT) for esophageal squamous cell carcinoma (ESCC) is beneficial in the setting of a complete pathological response. Rad51 expression affects both chemo- and radiosensitivity in many cancers; however, its role in ESCC is unclear.

**Methods:**

Rad51 expression was investigated by immunohistochemical staining with resected specimens in 89 ESCC patients who underwent surgery without preoperative therapy. The association with Rad51 and clinicopathological factors was assessed. The expression of Rad51 was also investigated in pretreatment biopsy specimens in 39 ESCC patients who underwent surgery after NACRT and compared with the pathological response to NACRT.

**Results:**

Lymph node metastasis was more frequently observed in Rad51-positive cases than negative cases (58.5 vs. 30.6 %, *P* = 0.0168) in patients treated with surgery alone. Disease-specific survival was decreased in Rad51-positive cases compared to Rad51-negative cases (5 year survival: 79.6 vs. 59.3 %, *P* = 0.0324). In NACRT patients, completed pathological responses were more frequently observed in Rad51-negative cases than in Rad51-positive cases (68.8 vs. 46.5 %, *P* = 0.0171).

**Conclusions:**

Rad51 expression in ESCC was associated with lymph node metastasis and poor survival. Additionally, Rad51 expression in pretreatment biopsy specimens was a predictive factor for the response to NACRT.

**Electronic supplementary material:**

The online version of this article (doi:10.1245/s10434-013-3220-2) contains supplementary material, which is available to authorized users.

Esophageal cancer, unlike other gastrointestinal malignancies, is extremely difficult to control with surgery alone.^1^ Neoadjuvant chemoradiotherapy (NACRT), frequently with cisplatin, is an important treatment strategy for advanced esophageal squamous cell carcinoma (ESCC); however, the clinical usefulness of NACRT for potentially resectable esophageal cancer remains controversial. In multiple meta-analyses, neoadjuvant treatment has been demonstrated to be superior to primary surgery in terms of local tumor control and disease-free survival.^2^–^4^ Other reports, however, have not demonstrated NACRT plus surgery to be superior to surgery alone.^5^–^7^ NACRT for esophageal cancer may also increase the risk of perioperative complications.^8^,^9^ Therefore, identification of molecular markers that predict the response to NACRT could potentially reduce perioperative complications by improving patient selection.

One predictive factor for chemoradiotherapy response in a variety of human cancers is Rad51.^10^,^11^ Rad51 is a key factor in homologous recombination.^12^ Overexpression of Rad51 decreases radiation sensitivity and confers resistance to DNA cross-linking agents such as cisplatin.^13^,^14^ We therefore hypothesized that Rad51 expression would predict the response to NACRT in ESCC.

The aims of this study were to evaluate the significance of Rad51 in ESCC and to correlate Rad51 expression in the pretreatment biopsy ESCC specimens with NACRT response.

## Materials and Methods

### Patients

Between July 1997 and March 2006, 89 patients (pT1–3, pN0–1, M0) with ESCC underwent esophagectomy without neoadjuvant therapy at the Department of Surgery and Science, Kyushu University Hospital, Fukuoka, Japan. The 78 male patients and 11 female patients ranged in age from 38 to 90 years (mean 63.3 years). Using the resected specimens from these patients, we evaluated the significance of the overexpression of Rad51 in ESCC.

For the NACRT group, patients were treated between 2003 and 2008. Thirty-nine patients (cT1–4, N0–1, M0) with ESCC underwent NACRT followed by esophagectomy: 22 patients at the Department of Surgery and Science, Kyushu University Hospital, and 17 patients at National Hospital Organization Kyushu Cancer Center, Fukuoka, Japan. For NACRT, 30–42 Gy of radiation was administered preoperatively to the primary tumor and metastatic lymph nodes. The chemotherapy regimen consisted of low-dose cisplatin and 5-fluorouracil (5-FU) (cisplatin: 5 mg/m^2^/day, 5-FU: 250 mg/m^2^/day, administered on weekdays, repeated every 3–4 weeks). Using pretreatment biopsy specimens in these patients, we compared the effectiveness of NACRT with the expression of Rad51.

### Immunohistochemistry

All surgically resected tumor specimens and biopsy specimens were fixed with 10 % formalin and embedded in paraffin. Four-micrometer sections were deparaffinized with xylene and rehydrated in a series of ethanols. Heat-induced epitope retrieval was performed in 0.1 M NaOH-citrate buffer (pH 7.0) for Rad51 immunostaining, and the samples were heated in an autoclave at 121 °C for 15 min. Endogenous peroxidase was blocked at room temperature using 3 % hydrogen peroxide in methanol for 30 min. After blocking with normal goat serum, slides were incubated with mouse monoclonal antibody against Rad51 (MS-988-P, NeoMarkers, Fremont, CA) using a 1:100 dilution of primary antibody at 4 °C overnight. After washing, the sections were treated for 60 min at room temperature with goat–anti-mouse immunoglobulin. Staining for Rad51 was completed using the streptavidin–biotin–peroxidase complex method with diaminobenzidine as a chromogen, and the slides were counterstained with hematoxylin. Positive staining was defined as a minimum of 10 % of the cancer cell nuclei showing positive nuclear staining.^15^ The tumors were staged according to the International Union Against Cancer’s tumor, node, metastasis (TNM) classification.^16^ A pathological complete response (pCR) was defined as no evidence of viable cancer cells in the primary regions; a pathological nonresponse was defined as viable cancer cells still observed.

### Assessment of Rad51 Staining in ESCC

Immunohistochemical staining was assessed for 89 samples from patients without preoperative therapy and 39 samples from patients who had undergone NACRT. Staining was scored under a light microscope by a pathologist (Nakashima Y) who was unaware of the clinical, pathological, and follow-up data. The concept of positive-cell index (PCI), indicating the proportion of positively stained tumor cells, was adopted for the analyses in this study. Rad51-positive staining was defined by identifying the optimal cutoff point. Qiao et al.^15^ have previously reported the optimal threshold required to separate prognostically. For this procedure, a PCI of 10 % was identified as the optimal cutoff. Cases whose immunohistochemistry (IHC) scores were less than 10 % were called “low-level expressers,” whereas those with IHC scores greater than 10 % were called “high-level expressers.” High-level expressers were defined as Rad51-positive staining cases, and low-level expressers were defined as negative staining cases.

In order to evaluate the heterogeneity of staining, we evaluated the area of Rad51 staining by dividing the tumor nest into three equal parts: the shallow level, the middle level, and the deep level of the invasive cancer.^17^ Homogenous staining was defined by identical staining patterns in all three parts of the tumor. If the staining pattern was homogenous, the Rad51 status in the biopsy specimen was considered to reflect the results in the surgical specimen.

### Statistical Analysis

The differences in distribution frequencies among the groups were evaluated using Fisher’s exact test or an unpaired *t*-test. The survival curves were plotted according to the Kaplan*–*Meier method and any differences were analyzed using the log-rank test. A multivariate analysis with Cox proportional hazards model was adopted to clarify the independent prognostic factors. Differences were considered to be significant if the *P* value was less than 0.05.

## Results

### Rad51 Expression in the Resected Specimens and Clinicopathological Factors in the Patients Who Underwent Surgery Without Preoperative Therapy

**Fig. 1:**
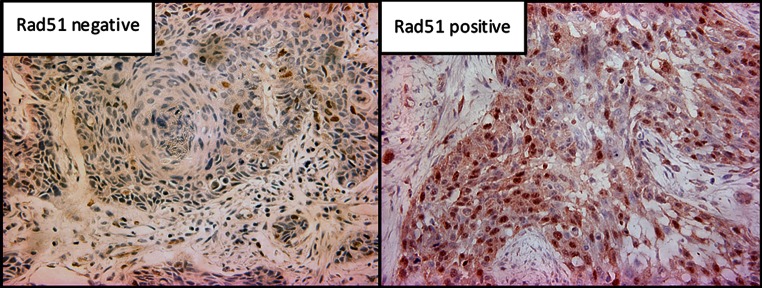
Immunohistochemistry for the detection of Rad51 in the resected specimens by esophagectomy without preoperative therapy (original magnification, ×200). Immunohistochemistry for Rad51 in ESCC resected specimens without preoperative therapy. Positive staining of Rad51 was present in 53 (59.6 %) cases and negative staining in 36 (40.4 %)

**Fig. 2:**
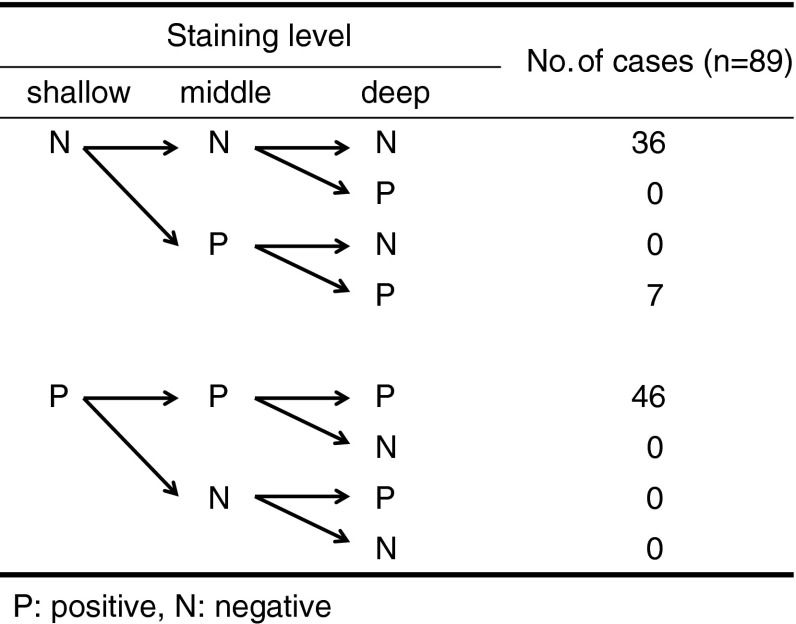
Rad51 staining patterns in surgical specimens. In 46 (86.7 %) cases of Rad51-positive staining in surgical specimens, a homogenous staining pattern was observed. In the seven cases with heterogeneous staining patterns, there was no expression of Rad51 in the shallow level, while positive expression was observed in the middle and deep levels of the tumor

Positive staining of Rad51 was observed in 53 (59.6 %) of 89 cases (Fig. 1). The patterns of Rad51 staining were homogenous in almost all specimens (Fig. 2). Additionally, 46 of 53 (86.7 %) cases without NACRT presented diffuse staining patterns from the shallow to deep levels of the tumor nest, with the expression pattern of Rad51 appearing homogenous. In the seven cases exhibiting heterogeneous staining patterns, there was no expression of Rad51 in the shallow level, while positive expression was observed in the middle and deep levels of the tumor.

**Fig. 3:**
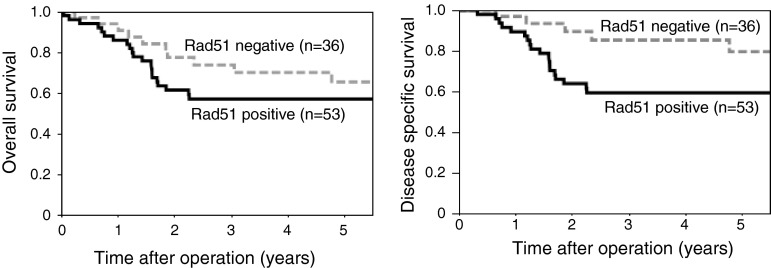
Survival for patients without preoperative chemoradiotherapy. Comparison of the 5-year OS rate and DSS rate between Rad51 positive and negative staining groups in ESCC without preoperative therapy. The difference in DSS was statistically significant (*P* = 0.0324)

There was a significant association between Rad51 expression and lymph node metastasis with node positive cases numbering 11 (30.6 %) and 31 (58.5 %) in the Rad51 negative and positive groups, respectively (Table 1, *P* = 0.0168). There were no significant associations between Rad51 expression and age, gender, or tumor location. There was no significant association between Rad51 expression and overall survival (Fig. 3; 5-year survival rates: 57.0 versus 65.4 %, for positive and negative, respectively; *P* = 0.1768). Rad51-positive cases had significantly poorer disease-specific survival compared to Rad51-negative cases (Fig. 3; 5-year disease-specific survival rates: 59.3 versus 79.6 % for positive and negative, respectively; *P* = 0.0324). In Rad51-negative cases, recurrence was less frequent than in Rad51-positive cases (Table 1; 9 of 36 cases versus 27 of 53 cases; *P* = 0.0171). In multivariate analysis, Rad51 was not an independent prognostic factor (Table 2, *P* = 0.5287), while lymph node metastasis was an independent prognostic factor (*P* = 0.0051).

**Table 1:** Rad51 expression in the resected specimen and clinicopathological factors in patients who underwent surgery without preoperative therapy

Factor	Rad51 negative	Rad51 positive	*P* value
	(*n* = 36)	(*n* = 53)	
Age (year)	62.3 ± 10.1	64.1 ± 9.9	0.5193
Sex			0.5144
Male	33 (91.7 %)	45 (84.9 %)	
Female	3 (8.3 %)	8 (15.1 %)	
Differentiation of ESCC			0.0999
Well	8 (22.2 %)	15 (28.3 %)	
Moderate	24 (66.7 %)	24 (45.3 %)	
Poor	4 (11.1 %)	14 (26.4 %)	
Location			0.1083
Upper	3 (8.4 %)	8 (15.1 %)	
Middle	21 (58.3 %)	19 (35.9 %)	
Lower	12 (33.3 %)	26 (49.1 %)	
Depth of invasion			0.1246
pT 1, 2	25 (69.4 %)	27 (50.9 %)	
pT 3	11 (30.6 %)	26 (49.1 %)	
Lymph node metastasis			0.0168
pN 0	25 (69.4 %)	22 (41.5 %)	
pN 1	11 (30.6 %)	31 (58.5 %)	
Lymphatic involvement			0.2794
Negative	22 (61.1 %)	25 (47.2 %)	
Positive	14 (38.9 %)	28 (52.8 %)	
Vascular involvement			1.0000
Negative	25 (69.4 %)	36 (67.9 %)	
Positive	11 (30.6 %)	17 (32.1 %)	
pStage			0.0905
I, II	30 (83.3 %)	35 (66.0 %)	
III	6 (16.7 %)	18 (34.0 %)	
Recurrence			0.0171
Negative	27 (75.0 %)	26 (49.1 %)	
Positive	9 (25.0 %)	27 (50.9 %)	

*ESCC* esophageal squamous cell carcinoma

**Table 2:** Multivariate analysis of Rad51 expression in the without NACRT

Factor	Object	Control	Odds ratio	95 % confidence interval	*P* value
Depth of invasion	T3/T4	T1/T2	0.92	0.77–2.12	0.3376
Lymph node metastasis	Positive	Negative	7.85	1.27–3.74	0.0051
Distant metastasis	Positive	Negative	0.05	0.19–4.16	0.8251
Recurrence	Positive	Negative	3.39	0.96–2.88	0.0654
Rad51	Positive	Negative	0.40	0.73–1.89	0.5287

*NACRT* neoadjuvant chemoradiotherapy

### Rad51 Expression in the Biopsy Specimens and Clinicopathological Factors in the Patients Who Underwent Surgery after NACRT

**Fig. 4:**
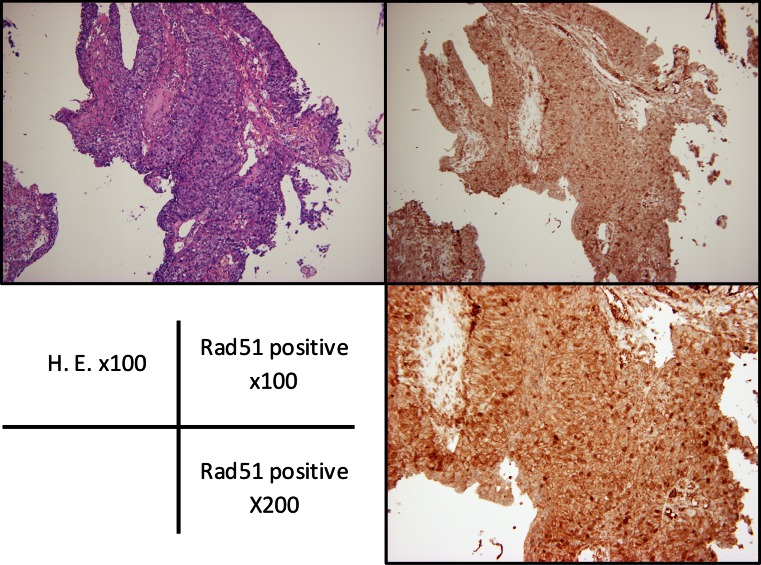
Immunohistochemistry for Rad51 in the biopsy specimens before preoperative therapy. Positive staining was present in 27 (69.2 %) cases, and 12 (30.8 %) cases were negative

Negative staining of Rad51 was observed in 12 (30.8 %) of 39 cases, while 27 cases were positive (69.2 %, Fig. 4). With respect to the efficacy of NACRT, seven of 39 cases (17.9 %) were histologically pCR. There were no significant associations between demographic and clinical factors, depth of invasion, lymph node metastasis, or TNM clinical stage (Table 3). Rad51 expression significantly predicted a response to NACRT; five (41.7 %) of 12 Rad51 negative cases were classified as pCR, while seven cases (58.3 %) were non-pCR patients. Twenty-five of 27 Rad51 positive cases (92.6 %) were non-pCR to NACRT with only two Rad51 positive cases (7.4 %) being classified as pCR (Table 4, *P* = 0.0197).

**Table 3:** Rad51 expression and clinical factors in patients with NACRT

Factor	Rad51 negative	Rad51 positive	*P* value
	(*n* = 12 %)	(*n* = 27 %)	
Age (year)	58.8 ± 12.3	62.5 ± 7.9	0.3071
Sex			0.6536
Male	9 (75.0 %)	23 (85.2 %)	
Female	3 (25.0 %)	4 (14.8 %)	
Differentiation of ESCC			0.6641
Well	2 (16.7 %)	8 (29.6 %)	
Moderate	8 (66.6 %)	16 (59.3 %)	
Poor	2 (16.7 %)	3 (11.1 %)	
Location			0.1850
Upper	3 (25.0 %)	10 (37.0 %)	
Middle	4 (33.3 %)	13 (48.2 %)	
Lower	5 (41.7 %)	4 (14.8 %)	
Depth of invasion			1.0000
cT 1, 2	2 (16.7 %)	4 (14.8 %)	
cT 3	10 (83.3 %)	23 (84.6 %)	
Lymph node metastasis			0.2694
cN 0	2 (16.7 %)	11 (40.7 %)	
cN 1	10 (83.3 %)	16 (59.3 %)	
cStage			0.6450
I, II	1 (8.3 %)	5 (18.5 %)	
III	11 (91.7 %)	22 (81.5 %)	

*NACRT* neoadjuvant chemoradiotherapy, *ESCC* esophageal squamous cell carcinoma

**Table 4:** Relationship of Rad51 expression and the responses of NACRT

Efficacy of NACRT	Expression of Rad51		*P* value
	Negative (*n* = 12)	Positive (*n* = 27)	
Non-pCR	7 (58.3 %)	25 (92.6 %)	0.0197
pCR	5 (41.7 %)	2 (7.4 %)	

*NACRT* neoadjuvant chemoradiotherapy, pCR pathological complete response

## Discussion

Overexpression of Rad51 has been observed in several cancers and may be involved in either the initiation or the progression of tumorigenesis.^18^,^19^ In non-small cell lung cancer (NSCLC), overexpression of Rad51 is related to decreased survival and increased tumor cell survival.^15^ Overexpression of Rad51 has been reported to correlate with histological grading of sporadic invasive ductal breast cancer, and is more frequently observed in advanced prostate cancer.^18^,^19^ These results suggest a relationship between Rad51 overexpression and more aggressive tumor behavior.

In ESCC, the significance of Rad51 overexpression is still unclear. In this study, high expression of Rad51 was associated with lymph node metastases in cases of esophageal cancer in which the patients had not undergone NACRT. However, the mechanism through which Rad51 expression affects the migratory ability of cancer cells has not been elucidated. Using canine adenocarcinoma metastatic models, it was demonstrated that Rad51 mRNA overexpression could be observed in metastatic lymph nodes.^20^ However, the details of the metastatic mechanisms mediating these effects are still unclear. In breast cancer, pancreatic cancer, soft tissue sarcoma, and non–small cell lung cancer, Rad51 overexpression was associated with poor prognoses, suggesting that Rad51 overexpression may enhance genetic instability and maintain DNA damage at a tolerable level to permit cell survival.^15^,^18^,^21^,^22^ The relationship between overexpression of double-stranded break (DSB) repair genes and the ability of tumor cells to undergo migration has not yet been elucidated. XRCC3, a DSB repair gene, was reported to be associated with increased tumor cell migration in breast cancer cells.^23^ Considering our results, which demonstrated that ESCC specimens with Rad51 overexpression often exhibited lymph node metastasis, further studies are needed to investigate the role of Rad51 using ESCC cell lines. On the basis of data from a tissue microarray, Li et al.^24^ reported that Rad51 was an independent prognostic factor in ESCC. In this study, Rad51 expression was investigated by conventional IHC using surgical resection and biopsy specimens. It is possible to investigate the entire cancer area using surgical and biopsy specimens by IHC; however, histological observation of the tumor nest is limited in tissue microarray analysis. Thus, differences in the evaluation methods for Rad51 expression may explain the inconsistencies between our study and previous studies.

In our study, some population bias was observed between the groups with and without NACRT; the NACRT group included more advanced cases of ESCC than the group without NACRT. Because there was a discrepancy in the association between lymph node metastasis and Rad51 expression in the groups with and without NACRT, we also analyzed Rad51 expression and clinicopathological factors, matching the staging of subjects. When limited to Stage I/II cases or Stage III cases, there were no significant relationships between lymph node metastasis and Rad51 expression, both in patients with and without NACRT, suggesting that the population bias resulted in variations in the association between lymph node metastasis and Rad51 expression (data not shown). Additionally, differences in staging methods, i.e. that patients’ backgrounds were based on pathological staging in the without NACRT group but on clinical staging in the with NACRT group, could explain the discrepancy in the association between lymph node metastasis and Rad51 expression in the three groups.

Our data indicated that Rad51 expression status in biopsy specimens could be a predictive factor for treatment efficacy of NACRT in ESCC. Because the Rad51 staining pattern was homogenous in the majority of cases, the expression pattern of biopsy specimens was considered to reflect the Rad51 expression status in the whole tumor nest. In assessment of the HER2 status of gastric cancer biopsies, the concordance rate of diagnosis between biopsy and surgical specimens was reported to be over 70 %.^25^,^26^ In this study, the concordance of Rad51-positive staining was 86.7 %, suggesting that Rad51 IHC results in biopsy specimens are useful as a predictive tool of Rad51-positive staining in surgical specimens, similar to the usefulness of HER2 status in biopsies of gastric cancer specimens.

Our data suggested that Rad51 is a useful predictive tool for NACRT in ESCC. However, Rad51 expression incompletely predicted the efficacy of NACRT, implying that the pathway is multifactorial. Thus, further studies are required in order to elucidate other mechanisms and markers that would allow us to predict the efficacy of NACRT.

DNA double strand breaks (DSBs), if unrepaired, are lethal to the tumor cell. Radiation and cisplatin trigger apoptosis in tumor cells by creating genetic instability through a DSBs mechanism.^27^ Rad51 plays an important role in the repair of DSBs through homologous recombination, thereby decreasing sensitivity to radiation and cisplatin. Radiation and CDDP inhibits cellular growth by inducing DNA DSBs.^28^–^30^ Cells can use DNA repair machinery to respond to the DNA damage. The levels of DNA repair proteins correlate with resistance to radiation and anticancer drugs in human cancer cell lines.^12^,^31^ Two pathways, homologous recombination and nonhomologous end joining, are used to repair DNA DSBs, and Rad51 is involved in the former process, homologous recombination. Recent evidence suggests that homologous recombination is involved in the repair of DNA DSBs generated by radiation and CDDP.^14^,^32^,^33^ Cancer cells may become resistant to radiation and CDDP by increasing the activity of homologous recombination repair machinery.^34^ On the other hand, 5-FU, an antimetabolic drug, exerts its antitumor effects through suppression of both DNA and RNA synthesis, pathways separate from CDDP or radiation. Because there are few studies of the direct relationship between DSB repair and 5-FU, further investigations with ESCC cells are required to elucidate the role of Rad51 in 5-FU sensitivity.^35^

In recent studies, down-regulation of Rad51 has been demonstrated to increase therapeutic sensitivities. In NSCLC, Tsai et al. reported that down-regulation of Rad51 using specific Rad51 small interfering RNA significantly increased cytotoxicity.^36^ Chan et al. 37 reported that down-regulation of Rad51 decreased homologous recombination, and increased sensitivity to the DNA cross-linking agents mitomycin C and cisplatin. Down-regulation of homologous recombination could result in low-fidelity DNA repair and have significant implications for response to therapy and genetic instability. Cancer cells may become resistant to cisplatin by increasing the activity of homologous recombination repair via Rad51 over expression. Down-regulation of Rad51 in ESCC may represent a novel therapeutic strategy to increase sensitivity for radiation and cisplatin chemotherapy.

In ESCC, radiation and cisplatin are the mainstays of treatment.^38^–^42^ NACRT with cisplatin results in significant down staging and induces pCR.^43^,^44^ A pCR to NACRT is critical for improving the survival of patients with ESCC.^45^ NACRT may, however, increase the incidence of postoperative complications.^46^ Thus, patient selection should identify those patients unlikely to benefit from NACRT. Although several other predictive factors have been reported, clear molecular prognostic factors are still needed to reduce the frequency of perioperative complications.^47^–^49^ On the basis of our results, Rad51 has great potential and warrants further investigation.

Considering our results, NACRT using a cisplatin/5-FU protocol is likely to fail when Rad51 overexpression is observed on a biopsy specimen. Use of NACRT for these patients may unnecessarily increase the rate of perioperative complications as there is no nonsurgical alternative to esophagectomy. In the current study, docetaxel, cisplatin and fluorouracil combination chemotherapy has been demonstrated to have activity in advanced and recurrent ESCC.^50^ Cetuximab may also have activity in ESCC.^51^,^52^ Rad51 targeted therapies may represent another novel therapeutic strategy.

In conclusion, Rad51 expression may predict NACRT response in ESCC. Rad51 expression may serve as a means by which to select patients for NACRT, thereby minimizing perioperative complications.

## Electronic supplementary material

Below is the link to the electronic supplementary material.

Supplementary material 1 (DOC 43 kb)

Supplementary material 2 (DOC 39 kb)

Supplementary material 3 (DOC 43 kb)

Supplementary material 4 (DOC 36 kb)
