# Supplementary material for: Rad51 Expression Is a Useful Predictive Factor for the Efficacy of Neoadjuvant Chemoradiotherapy in Squamous Cell Carcinoma of the Esophagus
Source: Ann Surg Oncol. 2013 Sep 25;21(2):597–604. doi: 10.1245/s10434-013-3220-2 (PMC3929771; doi:10.1245/s10434-013-3220-2)
Supplement: Supplementary file 1 — Supplementary material 1 (DOC 43 kb) [file 10434_2013_3220_MOESM1_ESM.doc]

Table S1: Rad51 expression in resected specimens and clinicopathological factors in pStage III patients who underwent surgery without pre-operative therapy.

| Factors | Rad51 negative  (n = 6) | |  | Rad51 positive  (n = 18) | | *P*-value |
| --- | --- | --- | --- | --- | --- | --- |
| Sex  Male  Female | 6  0 | (100)  (0) |  | 12  6 | (66.7)  (33.3) | 0.2770 |
| Differentiation of ESCC  Well  Moderate  Poorly | 0  6  0 | (0)  (100)  (0) |  | 6  7  5 | (33.3)  (38.9)  (27.8) | 0.0339 |
| Location  Upper  Middle  Lower | 0  3  3 | (0)  (50)  (50) |  | 3  4  11 | (16.7)  (22.2)  (61.1) | 0.3189 |
| Depth of invasion  pT = 1, 2  pT = 3 | 0  6 | (0)  (100) |  | 0  18 | (0)  (100) | - |
| Lymph node metastasis  pN = 0  pN = 1 | 0  6 | (0)  (100) |  | 0  18 | (0)  (100) | - |
| Lymphatic involvement  Negative  Positive | 1  5 | (16.7)  (83.3) |  | 3  15 | (16.7)  (83.3) | 1.0000 |
| Vascular involvement  Negative  Positive | 0  6 | (0)  (100) |  | 7  11 | (39.3)  (61.1) | 0.1300 |
| Recurrence  Negative  Positive | 3  3 | (50)  (50) |  | 2  16 | (11.1)  (88.9) | 0.0785 |
|  |  |  |  |  | (%) |  |
