# Supplementary material for: Rad51 Expression Is a Useful Predictive Factor for the Efficacy of Neoadjuvant Chemoradiotherapy in Squamous Cell Carcinoma of the Esophagus
Source: Ann Surg Oncol. 2013 Sep 25;21(2):597–604. doi: 10.1245/s10434-013-3220-2 (PMC3929771; doi:10.1245/s10434-013-3220-2)
Supplement: Supplementary file 2 — Supplementary material 2 (DOC 39 kb) [file 10434_2013_3220_MOESM2_ESM.doc]

Table S2: Rad51 expression and clinical factors in cStage III patients with NACRT.

| Factors | Rad51 negative  (n = 11) | |  | Rad51 positive  (n = 22) | | *P*-value |
| --- | --- | --- | --- | --- | --- | --- |
| Sex  Male  Female | 8  3 | (72.7)  (27.3) |  | 18  4 | (81.8)  (16.2) | 0.6610 |
| Differentiation of ESCC  Well  Moderate  Poorly | 2  8  1 | (18.2)  (72.7)  (9.1) |  | 7  12  3 | (31.8)  (54.6)  (13.6) | 0.5990 |
| Location  Upper  Middle  Lower | 3  4  4 | (27.2)  (36.4)  (36.4) |  | 10  9  3 | (45.5)  (40.9)  (13.6) | 0.2953 |
| Depth of invasion  cT = 1, 2  cT = 3 | 1  10 | (9.1)  (90.9) |  | 2  20 | (9.1)  (90.9) | 1.0000 |
| Lymph node metastasis  cN = 0  cN = 1 | 2  9 | (18.2)  (81.8) |  | 6  16 | (27.3)  (72.7) |  |
|  |  |  |  |  | (%) |  |
