# Supplementary material for: Rad51 Expression Is a Useful Predictive Factor for the Efficacy of Neoadjuvant Chemoradiotherapy in Squamous Cell Carcinoma of the Esophagus
Source: Ann Surg Oncol. 2013 Sep 25;21(2):597–604. doi: 10.1245/s10434-013-3220-2 (PMC3929771; doi:10.1245/s10434-013-3220-2)
Supplement: Supplementary file 3 — Supplementary material 3 (DOC 43 kb) [file 10434_2013_3220_MOESM3_ESM.doc]

Table S3: Rad51 expression in resected specimens and clinicopathological factors in pStage I/II patients who underwent surgery without pre-operative therapy.

| Factors | Rad51 negative  (n = 30) | |  | Rad51 positive  (n = 35) | | *P*-value |
| --- | --- | --- | --- | --- | --- | --- |
| Sex  Male  Female | 27  3 | (90)  (10) |  | 33  2 | (94.3)  (5.7) | 0.6553 |
| Differentiation of ESCC  Well  Moderate  Poorly | 8  18  4 | (26.7)  (60)  (13.3) |  | 9  17  9 | (25.7)  (25.7)  (48.6) | 0.4414 |
| Location  Upper  Middle  Lower | 3  18  9 | (10)  (60)  (30) |  | 5  15  15 | (14.2)  (42.9)  (42.9) | 0.3869 |
| Depth of invasion  pT = 1, 2  pT = 3 | 25  5 | (83.3)  (16.7) |  | 27  8 | (77.1)  (22.9) | 0.7658 |
| Lymph node metastasis  pN = 0  pN = 1 | 25  5 | (83.3)  (16.7) |  | 22  13 | (62.9)  (37.1) | 0.0958 |
| Lymphatic involvement  Negative  Positive | 21  9 | (70)  (30) |  | 22  13 | (62.9)  (37.1) | 0.6059 |
| Vascular involvement  Negative  Positive | 25  5 | (83.3)  (16.7) |  | 29  6 | (82.9)  (17.1) | 1.0000 |
| Recurrence  Negative  Positive | 24  6 | (80)  (20) |  | 24  11 | (68.6)  (31.4) | 0.3984 |
|  |  |  |  |  | (%) |  |
