# Supplementary material for: Rad51 Expression Is a Useful Predictive Factor for the Efficacy of Neoadjuvant Chemoradiotherapy in Squamous Cell Carcinoma of the Esophagus
Source: Ann Surg Oncol. 2013 Sep 25;21(2):597–604. doi: 10.1245/s10434-013-3220-2 (PMC3929771; doi:10.1245/s10434-013-3220-2)
Supplement: Supplementary file 4 — Supplementary material 4 (DOC 36 kb) [file 10434_2013_3220_MOESM4_ESM.doc]

Table S4: Rad51 expression and clinical factors in cStage I/II patients with NACRT.

| Factors | Rad51 negative  (n = 1) | |  | Rad51 positive  (n = 5) | |
| --- | --- | --- | --- | --- | --- |
| Sex  Male  Female | 1  0 | (100)  (0) |  | 5  0 | (100)  (0) |
| Differentiation of ESCC  Well  Moderate  Poorly | 0  0  1 | (0)  (0)  (100) |  | 1  4  0 | (20)  (80)  (0) |
| Location  Upper  Middle  Lower | 0  0  1 | (0)  (0)  (100) |  | 0  4  1 | (0)  (80)  (20) |
| Depth of invasion  cT = 1, 2  cT = 3 | 1  0 | (100)  (0) |  | 2  3 | (40)  (60) |
| Lymph node metastasis  cN = 0  cN = 1 | 0  1 | (0)  (100) |  | 5  0 | (100)  (0) |
|  |  |  |  |  | (%) |
